# Water-based medium-expansion foam depopulation of adult cattle

**DOI:** 10.1093/tas/txad065

**Published:** 2023-06-28

**Authors:** Vittoria M Capria, Andréia G Arruda, Ting-Yu Cheng, Magnus R Campler, Brad L Youngblood, Steven J Moeller, Andrew S Bowman, Justin D Kieffer

**Affiliations:** Department of Veterinary Preventive Medicine, College of Veterinary Medicine, The Ohio State University, Columbus, OH 43210, USA; Department of Veterinary Preventive Medicine, College of Veterinary Medicine, The Ohio State University, Columbus, OH 43210, USA; Department of Veterinary Preventive Medicine, College of Veterinary Medicine, The Ohio State University, Columbus, OH 43210, USA; Department of Veterinary Preventive Medicine, College of Veterinary Medicine, The Ohio State University, Columbus, OH 43210, USA; Department of Veterinary Preventive Medicine, College of Veterinary Medicine, The Ohio State University, Columbus, OH 43210, USA; Department of Animal Sciences, College of Food, Agricultural, and Environmental Sciences, The Ohio State University, Columbus, OH 43210, USA; Department of Veterinary Preventive Medicine, College of Veterinary Medicine, The Ohio State University, Columbus, OH 43210, USA; Department of Animal Sciences, College of Food, Agricultural, and Environmental Sciences, The Ohio State University, Columbus, OH 43210, USA

**Keywords:** cattle depopulation, foam depopulation, water-based medium-expansion foam

## Abstract

Current options for depopulation of adult cattle are limited, have logistic constraints, and may not be practical on a large scale. Aspirated water-based foam (**WBF**) has been shown to be successful in depopulating poultry and swine but has yet to be tested in cattle. WBF is advantageous because necessary equipment can be readily available, easy to use, and presents minimal personnel risk. With the use of a modified rendering trailer in a field setting, we evaluated the efficacy of aspirated WBF for depopulation of adult cattle. Water-based medium-expansion foam was added to the trailer holding cattle to a depth of approximately 50 cm greater than head height. The study was conducted as a gated design and the initial trial was conducted using six anesthetized and six conscious animals for verification of the process and followed by four replicates each containing 18 conscious cattle. A total of 84 cattle were used, with a subset (*n* = 52) implanted with subcutaneous bio-loggers that recorded activity and electrocardiograms. Cattle were loaded onto the trailer and three gasoline-powered water pumps delivered foam into the trailer followed by a 15-min foam dwell period. Average (± SD) time to completely fill the trailer with foam was 84.8 ± 11.0 s. No animal vocalizations were heard during foam application or the dwell period, and all cattle were confirmed dead upon removal from the trailer after 15 min of immersion. Necropsies of a subset of cattle revealed foam extending to at least the tracheal bifurcation in all cattle and distal to this level in 67% (8/12) animals. Time to cessation of movement, which served as a proxy for loss of consciousness, was 2.5 ± 1.3 min and time to cardiac death was 8.5 ± 2.5 min as determined by data from animals carrying subcutaneous bio-loggers. The results of this study indicate that WBF is a rapid and effective method for depopulation of adult cattle with potential advantages in speed and carcass handling and disposal over current methods.

## Teaser

Water-based foam is a rapid and effective method for depopulation of adult cattle in a field setting with potential advantages in speed and carcass handling and disposal over current methods.

## Introduction

Depopulation of cattle may need to be implemented for a variety of reasons, including foreign animal disease introductions, agroterrorism, intoxications or adulterations, radiologic or nuclear incidents, and natural disasters (Leary et al., 2019). An outbreak of a foreign animal disease such as foot and mouth disease would have devastating effects on the US livestock system (Paarlberg et al., 2002). Therefore, rapid and effective methods of depopulation to control disease spread and limit animal suffering are needed to be ready for implementation on short notice in these scenarios. Despite current guidelines for euthanasia and depopulation of cattle, challenges still exist in applying these methods to large populations in a timely manner. The goal of depopulation methods is to balance rapid crisis management and response with animal welfare while also considering the mental health of personnel tasked with depopulation as well as logistics (McReynolds and Sanderson, 2014; Shearer, 2018; Leary et al., 2019; Arruda et al., 2020).

The American Veterinary Medical Association (**AVMA**) Panel on Animal Depopulation emphasizes the importance of the following characteristics of a depopulation method: the ability to rapidly induce loss of consciousness followed by death with minimal pain or distress; reliability and irreversibility; personnel, animal, and environmental safety; psychological and emotional impacts on personnel; legal and religious requirements; and availability of agents as well as carcass processing and disposal options to handle the large volume (Leary et al., 2019). The current AVMA “Guidelines for the Depopulation of Animals” list humane slaughter, penetrating captive bolt (**PCB**), gunshot, and intravenous barbiturate administration as preferred methods for cattle depopulation (Leary et al., 2019). Slaughter is often an ideal method but may not be possible in scenarios where there is a food safety concern, animals are not fit for transport, the facility cannot handle the size or number of animals, or where transportation would cause disease spread, welfare concerns, or is not logistically possible. Penetrating captive bolt is effective but may not support rapid depopulation efforts when large numbers of animals are involved. Furthermore, PCB requires animal restraint and becomes time and labor intensive when applied in a chute as animals must be manually removed after depopulation (McReynolds and Sanderson, 2014). In addition, PCB guns must also be regularly cleaned, maintained, and periodically rested when used in quick succession (Leary et al., 2019). Gunshot applied to unrestrained cattle is problematic for the safety of personnel and animals in the area, poses welfare concerns for animals not instantly killed, and requires specialized training of personnel (McReynolds and Sanderson, 2014). In addition, both PCB and gunshot rely on accurate anatomical placement of the method. While AVMA euthanasia guidelines recommend application of a secondary method after PCB to ensure death, AVMA depopulation guidelines recognize that required application of a secondary method can add considerable time, supporting the alternative of confirmation of death on each animal and use of a secondary method as necessary (Leary et al., 2019; Leary et al., 2020). In all cattle depopulation scenarios, a secondary method should be available if needed but may add considerable time and effort if the primary method is not consistently effective. Intravenous barbiturates are effective but are controlled substances that are costly and require animal restraint as well as technical expertise to administer. In addition, barbiturates limit carcass disposal options as drugs are retained in the carcass after death. Therefore, large quantities of persisting barbiturates after a depopulation event and carcass disposal processes may result in contamination of the local environment and pose a risk to wildlife (Shearer, 2018; Leary et al., 2019). In all methods, carcass removal and disposal are limiting factors in the speed of depopulation and logistical concerns critical to depopulation success (McReynolds and Sanderson, 2014).

Due to limitations of current methods, there is a need for a rapid and effective depopulation method that allows for expeditious carcass disposal and considers personnel physical and mental well-being. Inhalation of aspirated water-based foam (**WBF**), causing asphyxiation by occluding the airway, has been extensively studied in poultry and is currently an AVMA-preferred method for floor-reared, confined poultry (Benson, et al., 2009; Caputo et al., 2012; Leary et al., 2019). In commercial poultry settings, WBF is considered a faster, more biosecure method of depopulation than carbon dioxide and does not negatively impact carcass composting (Benson, et al., 2009). Recent studies have also shown success in depopulating suckling, nursery, finisher, and cull swine using WBF in a bulk container or modified rendering trailer (Lorbach et al., 2021; Arruda et al., 2022; Kieffer et al., 2022). Some mentioned advantages of WBF over current depopulation methods include ease of use, minimal personnel risk, and availability of equipment as supplies needed for WBF depopulation already exist within the USDA National Veterinary Stockpile (USDA-APHIS). Thus, the goal of this study was to assess the efficacy of WBF applied in a modified rendering trailer as a depopulation method in adult cattle as measured by time to cessation of movement (**COM**) and terminal cardiac activity.

## Materials and Methods

### Ethics and Institutional Oversight

All animal experiments were performed under a protocol approved by The Ohio State University Institutional Animal Care and Use Committee (#2021A00000028). A PCB device was available after foam application if a secondary method of euthanasia was necessary. All cattle were housed and handled according to Guide for the Care and Use of Agricultural Animals in Research and Teaching (4th ed.).

### Animal Subjects and Study Design

#### Trial 1: small-scale field studies

Twelve multi-source cull dairy cattle, weighing an average of 614 kg (SD: 177 kg, range: 373 to 862 kg), were obtained for an initial pilot study. Trial 1A was conducted on six cattle that were anesthetized intramuscularly with ketamine (3.75 mg/kg Ketamine Hydrochloride, Covetrus NA, Dublin, OH), xylazine (0.375 mg/kg Anased LA, VetOne, Boise, ID), and butorphanol (0.0375 mg/kg Torbugesic, Zoetis, Kalamazoo, MI). A surgical plane of anesthesia was confirmed by observation of animals in lateral recumbency with fixed pupils before initiating foam application. Trial 1B was conducted on six conscious cattle. Necropsy of the respiratory tract was conducted on all 12 cattle.

In the pilot studies, all cattle were subcutaneously implanted with bio-loggers (DST centi-HRT ACT, Star Oddi, Gardabaer, Iceland) that recorded heart rate, electrocardiogram (**ECG**), and activity (accelerometer) at programmed 15-s intervals. The loggers were implanted over the right ventral thoracic body wall along the ribcage close to the heart. The subcutis was infiltrated with 2% lidocaine (VetOne) buffered 10:1 with 8.4% sodium bicarbonate (VetOne) and the surgical site was clipped and scrubbed using three rounds each of iodine scrub solution and 70% isopropyl alcohol. An approximately 3-cm incision was made over the lidocaine bleb and the area was bluntly dissected with surgical scissors and digital manipulation to create a pocket for the implant. The bio-logger was inserted into the pocket and the incision closed with 35W skin staples (AmerisourceBergen, Chesterbrook, PA). Loggers were programmed to take one measurement hourly for 12 h before initiation of foam to collect baseline data. During animal loading and foam application, all loggers were programmed to record every 15 s (the minimum recording interval allowed by the loggers).

#### Trial 2: large-scale field study

**Figure 1.**
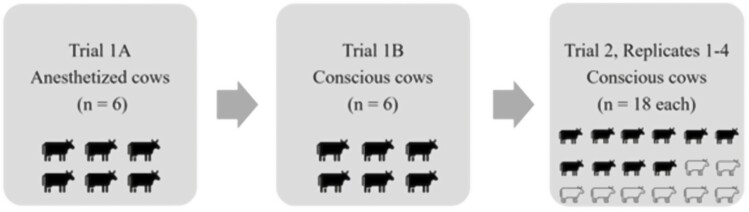
Chronological study progression demonstrating gated approach. Trial 1 included anesthetized adult cattle (Trial 1A, *n* = 6) then conscious adult cattle (Trial 1B, *n* = 6). All cattle in Trial 1 were implanted with bio-loggers. Trial 2 included four replicates of conscious cattle (*n* = 18 each) in which 10 of 18 cattle in each replicate were implanted with bio-loggers. Implanted cattle are denoted in black.

A total of 72 multi-source cull dairy and beef cattle were obtained and used in four replicates of the field study conducted over a 3-d period. Cattle were transported to the research site, off-loaded into the cattle holding facility, and offered ad libitum access to hay and water until randomized selection for inclusion in a replicate. The four replicates were conducted on conscious cattle in groups of 18 animals, providing a trailer space of 1.6 m^2^ space per animal. Within each replicate, a subset of cattle (10 of 18 in each replicate) was implanted with bio-loggers as described above (Figure 1). During the 3-d trial period, two replicates were conducted on day 1 and the other two were on days 2 and 3, respectively.

### Foam Production and Application

Cattle were loaded onto a 64.5 m^3^ (dimensions 12.2 m × 2.36 m × 2.24 m; length, width, height) custom-modified rendering trailer through the rear sliding door of the trailer using a single file ramp as described by Arruda et al. (2022). Once cattle were loaded, three individual foaming systems were used in parallel and consistently in all trials. A 1% foam-water solution was created by mixing PHOS-CHEK WD881 Class A foam concentrate (Perimeter Solutions, Rancho Cucamonga, CA, USA) in a 1,892-L water tank. The foam–water solution was pumped from the tank and delivered to the trailer as described by Arruda et al. (2022). With a foam expansion rate of approximately 40 to 50:1 foam to water, this was considered a medium-expansion foam. Bubble size was not measured. Foam was applied until it reached the top of the trailer, and the tarp was rolled over the open canopy section of the trailer top. The time to completely fill the trailer with foam was recorded and all cattle remained immersed for 15 min after the trailer was full. No top-ups of foam were required, and cattle were unable to be re-exposed to air once foaming was complete as the foam retained its level throughout the study period. After 15 min, the hydraulic lift on the trailer was engaged, the central cut gate was released, and cattle were offloaded by gravity from the rear of the trailer. Death was confirmed by the absence of corneal reflex, lack of respiration and body movement, and cardiac auscultation. Bio-loggers were retrieved for data download.

### Data Management and Analysis

The bio-loggers measured acceleration in three axes and normalized these values to the standard acceleration of gravity (9.8 m/s^2^). Forces causing acceleration of the logger other than gravity were calculated as a vectoral sum of acceleration and are reported as external acceleration (**EA**) in units of milligram. Natural forces causing acceleration act on the devices even when an animal is resting motionless, so the EA value is never zero. To discriminate between true animal motion and these natural forces, an EA threshold had to be determined as a cutoff value. This was done by first trimming EA data for each animal to include EA values from the start of foaming through the 15-min dwell period. The EA threshold value for each animal (*T*_EA_) was then calculated as the third quartile (EA_Q3_) plus 1.5 times the interquartile range (EA_Q3_ – EA_Q1_) obtained from EA values throughout the depopulation procedure (*T*_EA_ = EA_Q3_ + 1.5 × [EA_Q3_ – EA_Q1_]). EA data were processed using the device-associated software, Mercury v5.91 (Star Oddi).

**Figure 2.**
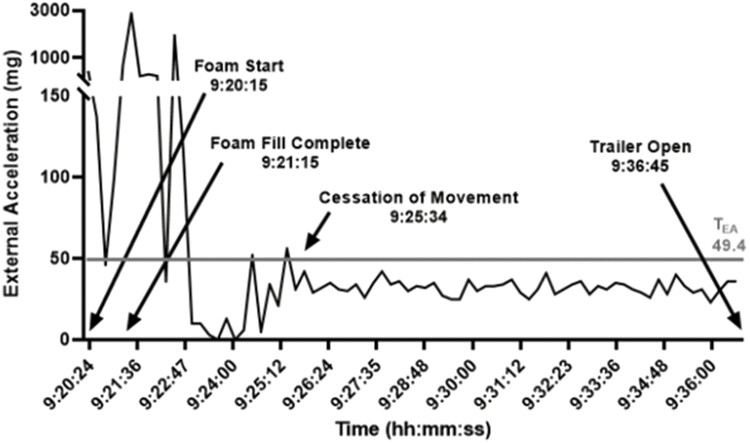
Example graph of external acceleration data over time during water-based foam application for an individual animal. Arrows indicate key timepoints during depopulation. The gray horizontal bar represents the external acceleration threshold (*T*_EA_) value for this animal. Notice that starting at 9:25:34 (considered the time of cessation of movement), there are no subsequent EA values above *T*_EA_ for the remainder of the time in foam.

As there are no guidelines or standards for establishing EA thresholds in cattle with this bio-logger, COM was defined as the first timestamp with an EA value below *T*_EA_ after which time no subsequent EA value was above *T*_EA_. For example, if an EA value was below *T*_EA_ at 3 min and all subsequent EA values were also below *T*_EA_, 3 min was considered the time to COM. A graphed example of EA output during WBF depopulation for an individual animal is shown in Figure 2 for added clarification. This method of data analysis was implemented as animal movement could not be confirmed visually through the foam. Descriptive statistics were used to report time to COM by replicating and collectively across all conscious implanted cattle. *T*_EA_ determination was not performed in anesthetized cattle due to very low EA values throughout the foaming and dwell periods.

Using the implanted Star Oddi bio-loggers, ECG waveforms were collected for later analysis and heart rate determination. Each implanted animal underwent waveform analysis to determine the time of cardiac death. Cardiac death was defined as the presence of a fatal arrhythmia (e.g., asystole, severe third-degree atrioventricular block, or ventricular fibrillation). ECG tracings were reviewed utilizing the associated Mercury software v5.99 and Pattern Finder software v1.17.0 (Star Oddi). The bio-loggers were programmed to record 3 s of ECG tracings every 15 s resulting in 12 s of tracings for analysis for each minute analyzed. To establish normal rate, rhythm, and appearance of ECG waveforms, baseline tracings were reviewed prior to foam initiation.

Statistical analyses were performed using R 4.2.2 (R Core Team, 2022). Time to COM and time to fatal rhythm were compared between Trial 1A and Trial 1B using a nonparametric Mann–Whitney *U* test. Because the heart rate was repeatedly measured within each animal throughout the depopulation process, the data were fitted into a linear model using generalized least squares with a first-order autoregressive variance-covariance structure. Variance components were estimated by restricted maximum likelihood. In addition, post hoc pairwise comparisons adjusted for multiplicity (Bonferroni correction) were performed to compare the heart rate of animals in two replicates at each time point of measurement. The assumption of normality was tested by examining the histogram of model residuals. For Trial 2, time to COM and time to fatal rhythm were compared among the four replicates using a nonparametric Kruskal–Wallis test, while post hoc pairwise comparisons were investigated using Dunn’s test (Dinno, 2015). The same modeling approach as in Trial 1 was used to investigate differences in the heart rate among the four replicates. Statistical significance was declared at *P* < 0.05.

## Results

### Trial 1: Small-Scale Field Study

**Figure 3.**
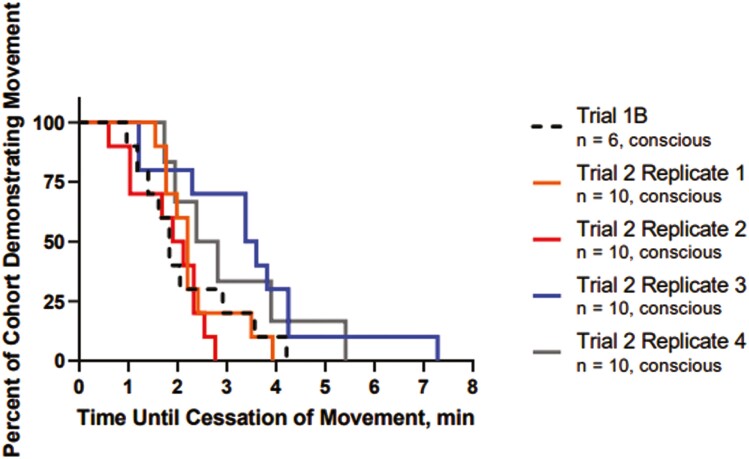
Kaplan–Meier survival curve showing probability of movement for 46 cattle depopulated using water-based foam in two cohorts based on bio-logger external activity. Trial 1B included six conscious, implanted cattle and all replicates in Trial 2 included 10 conscious, implanted cattle each.

**Figure 4.**
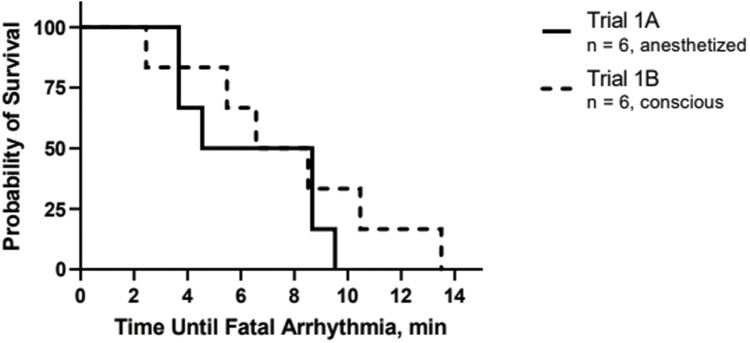
Kaplan–Meier survival curve showing probability of survival for 12 cattle depopulated using water-based foam in two cohorts based on bio-logger electrocardiograms. Trial 1A included six anesthetized, implanted cattle and Trial 1B included six conscious, implanted cattle.

The mean (± SD) time to completely fill the trailer with foam was 89.5 ± 4.9 s (Table 1). No animal vocalizations were heard during foam application or the dwell period in the pilot study and all animals were confirmed dead upon removal from the container at 15 min. The mean (± SD) time to COM from the end of the fill time in Trial 1B was 3.0 ± 1.4 min (Table 1, Figure 3). Focal necropsy of the upper respiratory tract of all animals in Trial 1 showed foam extending to the level of the tracheal bifurcation in all animals and distal to this level in 67% (8/12) animals. In one animal, intraluminal tracheal hemorrhage was observed. ECGs could be interpreted in at least 50% of time points assessed (Supplementary Figure S1a). There was no difference in average heart rate between animals in Trials 1A and 1B throughout the duration of the trial (*P* = 0.69). The mean (± SD) time of fatal rhythm from the end of the fill time was 7.2 ± 3.3 min in Trial 1 (Figure 4). There was no difference in time of fatal rhythm between Trial 1A and Trial 1B (7.8 ± 3.9 min and 6.5 ± 2.3 min, respectively; *P* > 0.05). Fatal arrhythmias (Supplementary Figure S3) which were identified included asystole (*n* = 1), third-degree atrioventricular block (*n* = 4), and ventricular fibrillation (*n* = 7).

**Table 1.** Trailer fill time and descriptive statistics for time to cessation of movement (COM)^1^ after water-based foam application in 46 conscious cattle as determined by activity data from implanted bio-loggers

Trial	Replicate	Time to COM (min)			
		Mean	Median	SD	Range
1A					
1B		3.0	2.6	1.4	1.7 to 5.4
2	1	3.5	3.5	1.7	1.2 to 7.3
	2	1.8	2.0	0.7	0.6 to 2.8
	3	2.4	2.2	0.8	1.6 to 3.9
	4	2.2	1.8	1.1	1.0 to 4.2
All	All	2.5	2.2	1.3	0.6 to 7.3

Trial 1A included six anesthetized cattle, Trial 1B included six conscious cattle, and Trial 2 included 10 conscious cattle in each replicate.

^1^External acceleration (EA) threshold value for each animal (*T*_EA_) calculated using the following formula: *T*_EA_ = EA_Q3_ + 1.5 × (EA_Q3_ − EA_Q1_), where EA_Q1_ and EA_Q3_ indicate the first and third quartile of EA measurements from the start of foaming through the 15 min dwell period. Time to cessation of movement (COM) defined as the first timestamp with an EA value below *T*_EA_ after which time no subsequent EA value was above *T*_EA_.

### Trial 2: Large-Scale Field Study

The mean (± SD) time to completely fill the trailer with foam was 82.5 ± 13.1 s. Data from all replicates are presented in Table 1. No animal vocalizations were heard during foam application or the dwell period in any replicates, and all animals were confirmed dead upon removal from the container at 15 min. The mean (± SD) time to COM across all replicates was 2.5 ± 1.3 min (Table 1) and there were no differences in average time to COM (*P* > 0.05).

**Figure 5.**
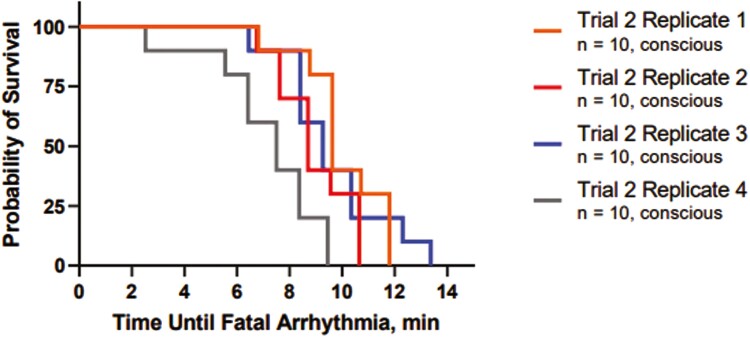
Kaplan–Meier survival curve showing probability of survival for 40 conscious cattle depopulated using water-based foam in four replicates based on bio-logger electrocardiograms. Each replicate included 10 conscious, implanted cattle.

ECG rhythms and tracings could be interpreted in more than 80% of cattle at baseline and from 3 min after application of foam through end of the 15 min of ECG monitoring. No ECGs could be interpreted at 2 min after initiation of foam application due to motion artifact and therefore were not analyzed. By 3 min after foam application, 85% of the ECGs could be interpreted. The number and percentage of ECGs which could be interpreted in each trial and replicated are presented in Supplementary Figure S1b. Overall, no difference in overall heart rate was found between the four replicates (*P* > 0.05). Cattle depopulated in Replicate 1 (98 ± 26 beats per minute (BPM)) had a higher average heart rate at baseline than cattle in Replicate 3 (57 ± 10 BPM, *P* = 0.003) and Replicate 4 (67 ± 5 BPM, *P* = 0.0208). Cattle depopulated in Replicate 1 (105 ± 41) had a higher average heart rate at 3 min after initiation of foam application than Replicate 4 (73 ± 23, *P* = 0.04). Cattle depopulated in Replicate 2 (95 ± 40) had a higher average heart rate at 4 min after the initiation of foam application than Replicate 3 (65 ± 32, *P* = 0.041). There was no difference in average heart rate between animals in each replicate beginning at 5 min after initiation of foam application through the end of the monitoring period (*P* > 0.05). The mean (± SD) time of fatal rhythm from the end of the fill time was 9.0 ± 2.1 min (Figure 5). Cattle depopulated in Replicate 1 had a significantly shorter average time to fatal rhythm than animals in Replicate 4 (7.2 ± 2.1 min and 10.0 ± 1.6 min, respectively; *P* = 0.003). There were no significant differences between animals in any of the other replicates with respect to average time to fatal rhythm. Fatal arrhythmias (Supplementary Figure S3) which were identified included asystole (*n* = 20) and third-degree atrioventricular block (*n* = 20).

## Discussion

We sought to assess the efficacy of WBF as a depopulation method for adult cattle and our results indicate that WBF causes irreversible unconsciousness followed by 100% mortality after 15 min of foam immersion. In this study, COM and cardiac death were achieved on average after 2.5 and 8.5 min, respectively, after being immersed in WBF. These results are consistent with WBF application in other species from the time of foam fill completion. In poultry, average time to COM ranged from 0.8 to 1.8 min and average terminal cardiac activity was approximately 1.1 min (Dawson et al., 2007). In studies of WBF application in Pekin ducks, average time to COM ranged from 3.3 to 5.9 min and average terminal cardiac activity ranged from 4.3 to 5.0 min (Benson, et al., 2009; Caputo et al., 2012). In swine, WBF has been reported to cause COM on average in 2.1 to 2.2 min in cull sows, 1.4 to 1.8 min in finisher pigs, and 0.8 to 2.8 min in suckling piglets (Lorbach et al., 2021; Arruda et al., 2022; Kieffer et al., 2022). Average terminal cardiac activity after WBF has been reported as 8.7 min in cull sows and 7.3 min in nursery pigs (Arruda et al., 2022). Given that WBF is effective in multiple species, its versatility could be a suitable and universal option for farms housing a variety of species. For example, a foot-and-mouth disease outbreak on a multi-species farm would enable depopulation of all animals using WBF instead of having to apply varying methods to each species.

Advantages of WBF reported in other species were also realized in the present study. Application of WBF was rapid under the production process described, and time to COM indicated a rapid loss of consciousness in cattle. The time to fill a 64.5-m^3^ trailer using three simultaneous pumps was 93 s or less, noting that animal size and loading density influence fill time, we demonstrated effective depopulation of up to 18 cattle at once. Fifteen minutes of foam immersion was sufficient to cause 100% mortality; therefore, the present study indicates the capability to depopulate a group of cattle, from initiation of foam application to end of a 15-min dwell period, in less than 17 min using a single deck containment trailer and sufficient pump capacity. While we did not measure animal loading time, we can subjectively note that loading time was consistent with expectations of loading a semi-trailer via a cattle alley and loading chute within a typical cattle handling facility. In the present study, we found that WBF presented relatively low safety risks to personnel involved in depopulation efforts as the 1% foam–water mixture is nontoxic and personal protective equipment was worn while applying WBF to cattle and handling the concentrate solution. We also concluded, from a caretaker standpoint, that the applied WBF setup using a modified rendering trailer was a safe depopulation method for cattle. While no behavioral analyses were conducted, limited escape attempts were observed until the foam reached shoulder height of the cattle and staff safely monitored the process from an elevated viewpoint and would at no point be in harm’s way. Finally, the robust solid wall design of the trailer itself would be unbreachable by cull cattle of adult size and pose no danger for any caretakers or staff on the ground.

While gross anatomical pathology of the respiratory tract was only observed in one animal, foam exposure time was relatively short. The intraluminal tracheal hemorrhage seen in one animal was likely due to mucosal capillary leakage, which is a common finding in asphyxiated animals, including poultry euthanized with WBF (Dawson et al., 2006; Benson, et al., 2007).

Disposal of carcasses in a timely manner and the ability to match the rate of disposal to the rate of depopulation have been identified as a critical factor in depopulation efforts (McReynolds and Sanderson, 2014). The primary advantage of WBF applied in a modified rendering trailer relates to carcass disposal as once depopulation is complete, carcasses can be driven to the site of disposal and are quickly and easily removed via the hydraulic lift feature of the trailer. During this process, carcasses, waste, and the foam solution are well-contained in the trailer which may help limit spread of infectious diseases as personnel do not have to handle carcasses after depopulation.

Another advantage of this WBF setup is that the foam-generating equipment is mobile, easily obtained, and could be quickly transported to the needed location and assembled, while being compatible with various cattle housing systems and production settings. Under true field depopulation conditions, the capacity and number of depopulation events possible in a given time frame will be determined by the size of the vessel, time to load, number of pumps and volume capacity to deliver WBF, delivery and storage rate of water, dwell time, time to unload, and the round-trip time to a disposal site.

Psychological well-being of personnel involved in depopulation efforts has been identified as an important factor to consider when selecting a depopulation method (McReynolds and Sanderson, 2014; Shearer, 2018; Leary et al., 2019; Arruda et al., 2020). Studies from the 2001 foot-and-mouth disease outbreak in the United Kingdom found that those involved in animal depopulation had negative emotional impacts that included distress, bereavement, and fear (Mort et al., 2005). Surveys of veterinarians involved in swine depopulation efforts associated with the COVID-19 pandemic showed high levels of burnout and emotional trauma (Baysinger and Kogan, 2022; Bussolari et al., 2022; Kollias et al., 2023). While depopulation is always likely to cause some level of emotional stress on personnel, physical methods of euthanasia have been shown to be associated with higher rates of burnout, compassion fatigue, and poor professional quality of life as compared to nonphysical methods (LaFollette et al., 2020). Future studies should assess personnel perceptions regarding WBF in cattle as compared to other available depopulation methods.

ECG assessments have been extensively utilized as clinical indicators of death in cattle. In a study of a pneumatic captive bolt device euthanasia of feedlot calves, heartbeat was detected via stethoscope up to 14.4 min, and cardiac activity detectable via ECG up to 14.8 min after application with a mean time to cardiac death of 8.4 min (Derscheid et al., 2016). In a study of beef calves killed by PCB, cardiac electrical activity persisted for an average of 8.6 min but persisted in one animal for over 15 min. However, 95% of animals showed cardiac standstill within 15 min. Importantly, this study highlighted that electrical cardiac death occurred after clinically detectable cardiac death in 70% of animals (Dewell et al., 2015). This is likely due to electromechanical dissociation or pulseless electrical activity. In this study, the average time to development of a terminal arrhythmia was 8.5 min after initiation of the foam application. All cattle developed a fatal arrhythmia within 15 min of beginning foam application. The heart is only capable of generating functional or mechanical activity with electrical stimulation but is capable of generating electrical activity that does not contribute to physiologic or mechanical function. It is probable that animals are not generating functional cardiac activity before electrical activity has ceased. Evaluation of ECG has previously been used to assess slaughter methods in cattle. In one study, both penetrating and nonpenetrating captive bolt resulted in apparently normal ECG immediately after stunning with a heart rate of 120 BPM (Blackmore, 1989). In the present study, heart rates in Trial 1A cattle remained relatively unchanged following application of foam, which is likely due to anesthesia. In Trial 1B, there was a noted increase in heart rate 2 min after initiation of foam application. It is likely this increase in heart rate was associated with a physiologic response to hypoxia and/or is a stress response. No animals in Trial 2 had interpretable ECGs at 2 min following foam application. The ECG recordings for these animals were likely influenced by animal movement resulting in excessive noise in the recordings. This movement may have been due to terminal convulsions or escape attempts, but this information cannot be known as cattle were unable to be visualized through the foam. Beginning at 3 min after initiation of foam application, the heart rate in Trial 1B animals was elevated when compared to baseline but had returned to baseline values by 4 min after initiation of foam application. This same trend was identified in Trial 2 animals and is likely attributed to a hypoxia or stress related to foam application.

One limitation of this study is that cattle could not be directly observed through the foam to confirm activity output from the implanted bio-loggers. Therefore, we needed to develop a methodology to determine which EA values represented true animal movement. The method used was based on the calculation of outliers for skewed data as our dataset included periods of high EA values during the initial phase of the foaming process (Carling, 2000). This method determined two animals with times to COM of 5.4 and 7.3 min based on EA values within less than 2 units of *T*_EA_. These times were substantially longer than all other times to COM (≤4.3 min). Since it is unlikely for animals to remain conscious and active at this point, the movement could be caused by minor postmortem spasms or external forces as the EA values were slightly above the cutoffs. It is also noteworthy that these animals were in Trial 1B and Trial 2 Replicate 1, both of which had the longest times to COM. While these long times to COM may represent external forces acting upon the animal or unconscious animal movement, an inability to definitively determine animal movement meant we used the longest possible time to COM. While these devices have not been used to determine COM in cattle before, a previous study has utilized the same devices to monitor activity in cattle, assessing relative activity levels but did not need to categorize EA values in a binomial fashion (Palacios et al., 2021). These devices have also been validated to monitor activity of Atlantic salmon (Zrini and Gamperl, 2021). External accelerometers have been used extensively to determine COM in poultry and swine as a proxy for electroencephalography (**EEG**) and therefore brain death during various euthanasia methods, including WBF (Dawson et al., 2007; Benson, et al., 2009; Caputo et al., 2012; Lorbach et al., 2021; Arruda et al., 2022). While accelerometers are easier to implement than EEG, it is established that unconsciousness occurs before convulsions in both poultry and cattle (Dawson et al., 2007; Leary et al., 2019; Collins et al., 2020). In a study that utilized EEG and external accelerometers in calves euthanized via captive bolt, unconsciousness occurred in 0 s, but convulsions lasted up to 13.8 min (Collins et al., 2020). A study in WBF-depopulated nursery pigs demonstrated using EEG that unconsciousness occurred in 1.9 min and brain death in 3.1 min, supporting that time to COM is an overestimate of time to loss of consciousness (Korenyi-Both et al., 2022). Therefore, it is likely that COM overestimates the time to unconsciousness in cattle and represents a conservative estimate of unconsciousness, which is the parameter most relevant to animal welfare during euthanasia or depopulation. Electroencephalography remains the most objective method to assess unconsciousness but is difficult to obtain in adult cattle due to the thickness of their skull (Verhoeven et al., 2015). Future studies should assess efficacy of WBF in calves where EEG could be more easily applied (Bager et al., 1992; Collins et al., 2020).

## Conclusions

The results of the present study demonstrated that WBF is a promising option for depopulation of adult cattle in a field setting. The ease and speed with which it can be applied to groups of cattle in a modified rendering trailer make it a practical method that could be added to our available depopulation techniques. While we know a 15-min dwell period is 100% effective in causing mortality in adult cattle, additional research is needed to determine at which timepoint animals become nonrecoverable. This information will enable the dwell period to be shortened to the minimum time necessary and therefore speed depopulation efforts. In addition, the broad methodological framework and multi-special application may enable WBF to function as overarching emergency depopulation method for farms housing multiple species. Additional research is also needed to validate WBF in calves, to determine personnel perceptions of WBF application as compared to other methods in cattle, and to understand potential impacts on composting or other methods of animal carcass disposal.

## Supplementary Material

txad065_suppl_Supplementary_Figure_S1A

txad065_suppl_Supplementary_Figure_S1B

txad065_suppl_Supplementary_Figure_S2

txad065_suppl_Supplementary_Figure_S3

txad065_suppl_Supplementary_Materials
