# Supplementary figures and images for: Water-based medium-expansion foam depopulation of adult cattle
Source: Transl Anim Sci. 2023 Jun 28;7(1):txad065. doi: 10.1093/tas/txad065 (PMC10321402; doi:10.1093/tas/txad065)

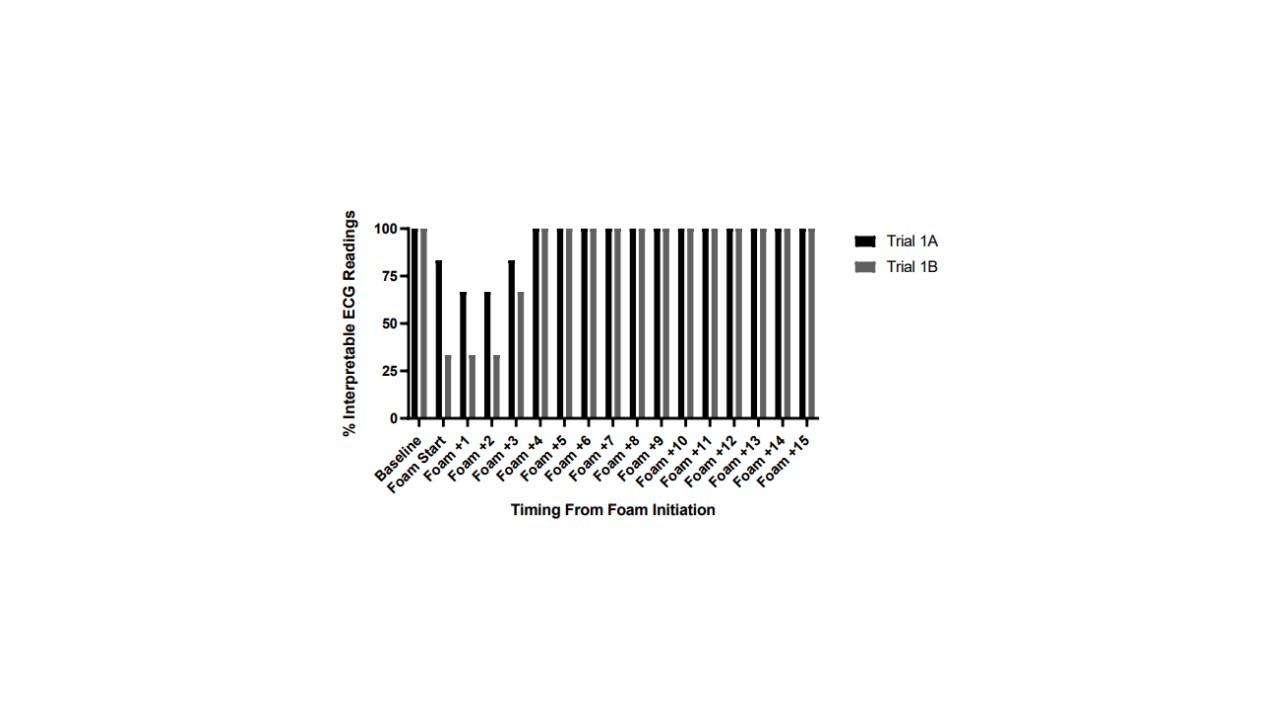

Supplement: txad065_suppl_Supplementary_Figure_S1A [file txad065_suppl_supplementary_figure_s1a.jpeg]

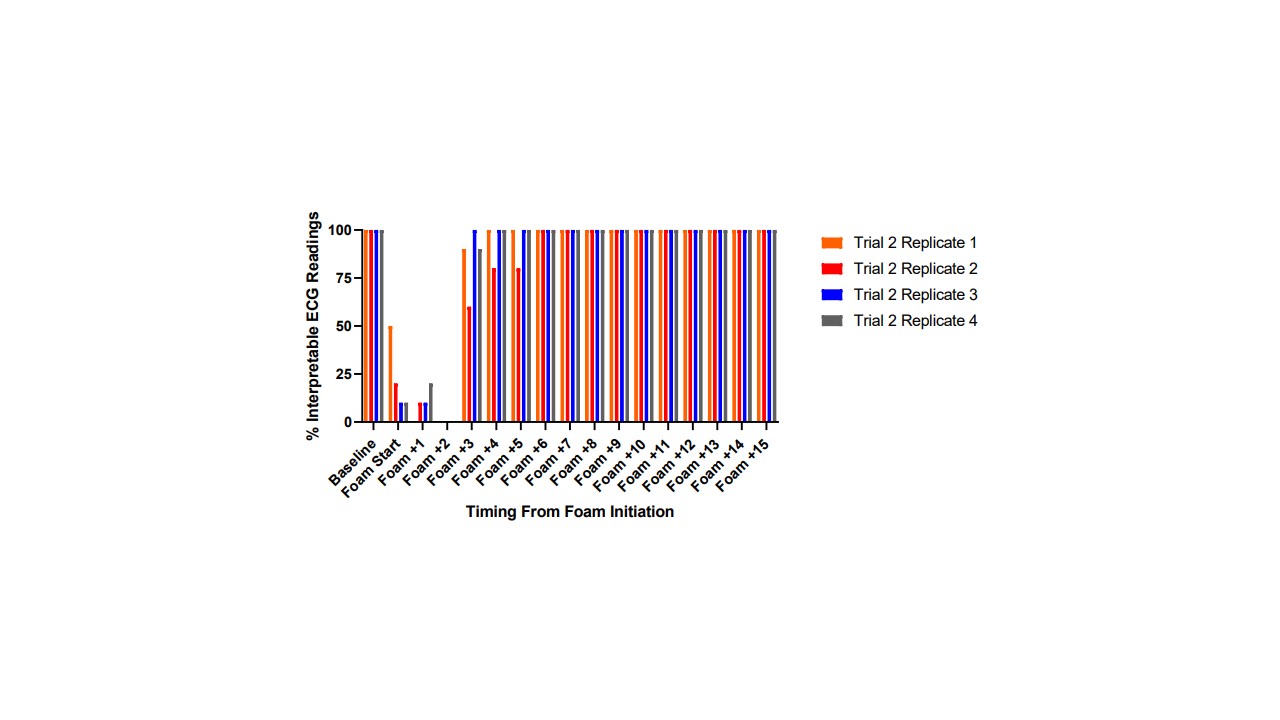

Supplement: txad065_suppl_Supplementary_Figure_S1B [file txad065_suppl_supplementary_figure_s1b.jpeg]

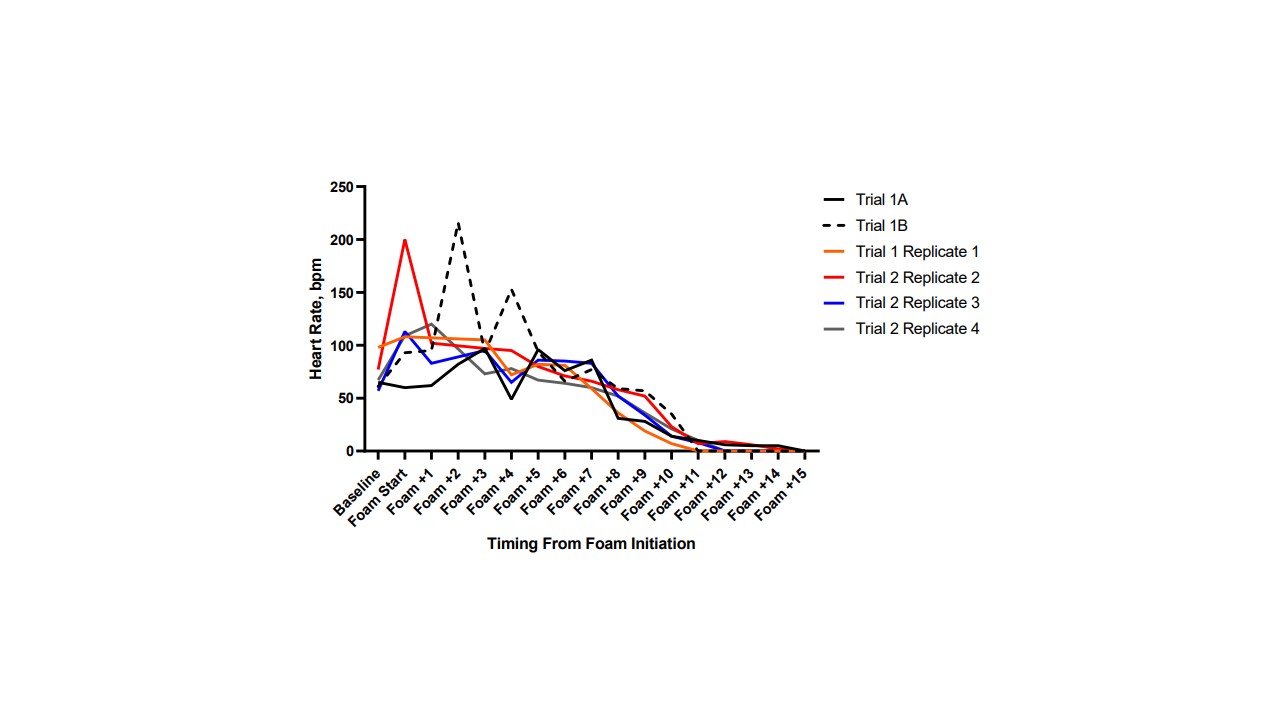

Supplement: txad065_suppl_Supplementary_Figure_S2 [file txad065_suppl_supplementary_figure_s2.jpeg]

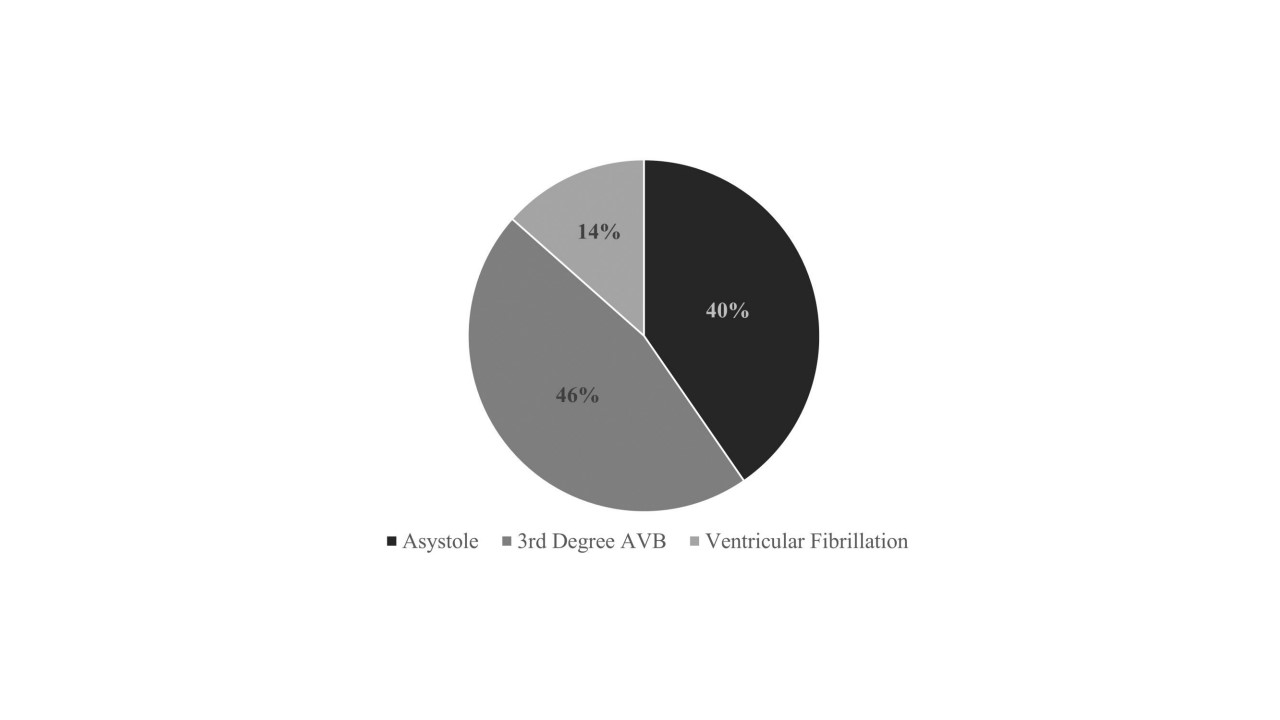

Supplement: txad065_suppl_Supplementary_Figure_S3 [file txad065_suppl_supplementary_figure_s3.jpeg]
