# Supplementary material for: Water-based medium-expansion foam depopulation of adult cattle
Source: Transl Anim Sci. 2023 Jun 28;7(1):txad065. doi: 10.1093/tas/txad065 (PMC10321402; doi:10.1093/tas/txad065)
Supplement: txad065_suppl_Supplementary_Materials [file txad065_suppl_supplementary_materials.docx]

**Supplementary Materials**

Figure 1a: Percentage of ECG readings which could be interpreted in Trial 1 over time (e.g., Foam +1 indicates 1 minute following initiation of foam). Trial 1A included 6 anesthetized cattle and Trial 1B included 6 conscious cattle.

Figure 1b: Percentage of ECG readings which could be interpreted in Trial 2 over time (e.g., Foam +1 indicates 1 minute following initiation of foam). All replicates included 10 conscious cattle each.

Figure 2. Heart rate as beats per minute in ECG recordings which could be interpreted over time (e.g., Foam +1 indicates 1 minute following initiation of foam application). Trial 1A included 6 anesthetized cattle, Trial 1B included 6 conscious cattle, and all replicates in Trial 2 included 10 conscious cattle each.

Figure 3. Percent of fatal arrhythmias identified in ECG recordings which could be interpreted in both trials (n=52).
